# An implementation model for scaling up oral pre-exposure prophylaxis in Kenya: *Jilinde* project

**DOI:** 10.12688/gatesopenres.13342.1

**Published:** 2021-07-27

**Authors:** Daniel Were, Abednego Musau, Mary Mugambi, Marya Plotkin, Mark Kabue, Griffins Manguro, Steven Forsythe, Robert Glabius, Eunice Mutisya, Manya Dotson, Kelly Curran, Jason Reed

**Affiliations:** 1 Jhpiego, Kenya, Nairobi, 00800, Kenya; 2 Division of National AIDS and STI Control Program, Nairobi, 00202, Kenya; 3 Jhpiego, USA, Baltimore, Maryland, 21231, USA; 4 International Center for Reproductive Health, Kenya, Mombasa, 80103, Kenya; 5 Avenir Health, Glastonbury, Connecticut, 06033, USA; 6 Population Services Kenya, Nairobi, 00400, Kenya

**Keywords:** Oral PrEP, scale up, demand, supply, ownership, learning laboratory, key populations, adolescent girls and young women

## Abstract

Oral pre-exposure prophylaxis (PrEP) is an efficacious way to lower the risk of HIV acquisition among high-risk individuals. Despite the World Health Organization’s 2015 recommendation that all persons at substantial risk of HIV infection be provided with access to oral PrEP, the rollout has been slow in many low- and middle-income countries. Initiatives for national rollout are few, and subtle skepticism persists in several countries about the feasibility of national PrEP implementation. We describe the conceptual design of the *Jilinde* project, which is implementing oral PrEP as a routine service at a public health scale in Kenya. We describe the overlapping domains of supply, demand, and government and community ownership, which combine to produce a learning laboratory environment to explore the scale-up of PrEP. We describe how *Jilinde* approaches PrEP uptake and continuation by applying supply and demand principles and ensures that government and community ownership informs policy, coordination, and sustainability. We describe the “learning laboratory” approach that informs strategic and continuous learning, which allows for adjustments to the project. *Jilinde’s* conceptual model illustrates how the coalescence of these concepts can promote scale-up of PrEP in real-world conditions and offers critical lessons on an implementation model for scaling up oral PrEP in low- and middle-income countries.

## Disclaimer

The views expressed in this article are those of the author(s). Publication in Gates Open Research does not imply endorsement by the Gates Foundation.

## Introduction

The global HIV response has seen positive developments over the past two decades, characterized by a decline in both the number and rate of new HIV infections and AIDS-related deaths ^1^. However, AIDS-related sequelae still contribute a considerable burden to morbidity and mortality in countries in sub-Saharan Africa (SSA), which continue to disproportionately bear the brunt of the epidemic and accounted for the majority of the new HIV infections in 2019 ^1^. While 72% of new HIV infections in east and southern Africa occurred among the general population, nearly 30% of the overall new HIV infections were attributed to adolescent girls and young women (AGYW) aged 15–24 years ^2^. In the same region, key populations (KP) including female sex workers (FSW), men who have sex with men (MSM), persons who inject drugs (PWID), transgender individuals, and their sexual partners contribute the remaining 28% of new HIV infections ^2^.

Kenya, one of the high HIV burden countries in SSA, has made considerable progress in its HIV response. The national HIV prevalence declined from 5.6% in 2012 to 4.9% in 2018, and further reductions in new HIV infections occurred among adults (106,000 to 36,000) within the same period ^3, 4^. Despite these gains, HIV prevalence and incidence remain high in certain geographic pockets and among specific populations. Kenya has a geographically disproportionate epidemic that is mainly concentrated around the Lake Victoria region. In 2017, half of Kenya’s estimated new HIV infections among adults occurred in six of Kenya’s 47 counties, four of them in the Lake Victoria region ^5^.

A 2008 study on the modes of HIV transmission reported that KP contribute approximately one-third of new infections in Kenya ^6^, and this has persisted to date. More recent estimates show that AGYW aged 15–24 years accounted for a third of all new adult HIV infections in 2017 ^5^. Furthermore, AGYW are four times more likely to acquire HIV infection compared to their male counterparts ^7^. Substantial work remains to lower HIV transmission rates among these groups, who have a higher prevalence and incidence than the general population, as this high HIV prevalence and incidence threatens the sustainability of gains made in the general population.

To address these disparities in prevalence and incidence, Kenya has mounted a robust HIV response modeled on the combination prevention approach, with geographic and population prioritization ^8^. However, several gaps hinder the optimization of this strategy including a large number of HIV-infected individuals who do not know their HIV status and therefore are not on HIV treatment. Similarly, despite wide availability of condoms, many high-risk individuals do not use them consistently ^9– 11^. While voluntary medical male circumcision will likely eventually reduce overall community HIV prevalence in Kenya, it does not provide direct prevention benefits for AGYW and FSW, or MSM at risk from engaging in receptive anal sex. Further, while treatment as prevention, treatment of sexually transmitted infections (STIs), and post-exposure prophylaxis ^12– 15^ are all available to KP and AGYW, the use of most of these interventions is less than optimal. This is mainly due to contextual barriers including gender-based violence, stigma and discrimination, legal obstacles, gender and cultural norms, and access barriers ^16– 19^. These gaps signal that a substantial proportion of individuals engaging in high-risk sexual encounters require a reliable prevention alternative.

Oral pre-exposure prophylaxis (PrEP) has proven to be highly efficacious at lowering the risk of HIV transmission when consistently taken daily ^20^ as well as intermittently among a sub-group of MSM ^21^. In September 2015, the World Health Organization recommended that people at substantial risk of HIV infection should be offered oral PrEP as an additional prevention choice as part of a comprehensive prevention services package ^22^. After the release of these recommendations, UNAIDS set an ambitious target of starting 3 million people on oral PrEP by 2020 ^23^. Realization of this target was dependent on rapid scale-up of oral PrEP across multiple countries, especially those most affected by the epidemic. By December 2020, an estimated 930,000 people had received PrEP ^24^. The availability of oral PrEP in low- and middle-income countries has largely been limited to research initiatives and demonstration projects, with few examples of national rollout ^25^. In spite of the documented benefits and global recommendations, many countries are still skeptical about the feasibility of national rollout of oral PrEP ^26, 27^. This skepticism is fueled by a gap in evidence on the feasibility and effectiveness of population-level PrEP interventions in low- and middle-income countries. This paper describes the conceptual design of an oral PrEP scale-up model implemented by the *Jilinde* project in Kenya.

### The *Jilinde* project

The *Jilinde* (Bridge to Scale) project is designed as a “learning laboratory” to implement oral PrEP at scale, while simultaneously deriving lessons to understand the barriers and enablers to PrEP scale-up. *Jilinde* is a Kiswahili word that means “protect yourself.” This five-year (2016–2021) project is implemented in Kenya by a consortium of five partners that includes: Jhpiego, the National AIDS and STIs Control Program (NASCOP), International Center for Reproductive Health - Kenya, Population Services Kenya, and Avenir Health.

### The setting

**Figure 1.**
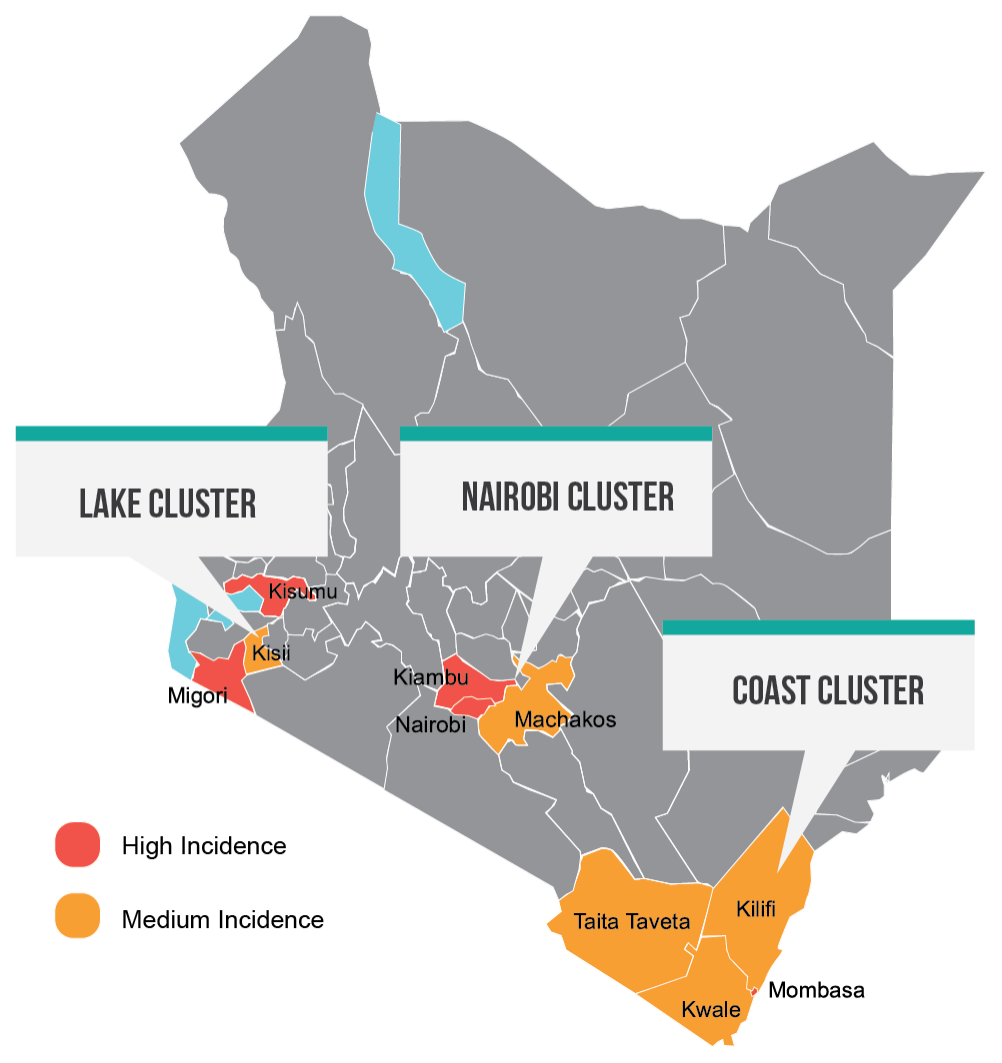
Geographic coverage of the *Jilinde* project based on HIV incidence.

The project is implemented in 10 priority counties in Kenya, which were selected according to the severity of the HIV epidemic as illustrated in Figure 1.

Data on the geographic incidence and burden of HIV was used to prioritize intervention counties based on an incidence threshold of 0.081% - 0.13% for medium incidence and 0.131% or more for high incidence, per 100 person-years ^28^. Consequently, *Jilinde* is implemented in five high-incidence counties (Kisumu, Migori, Nairobi, Kiambu, and Mombasa) and five medium-incidence counties (Machakos, Kisii, Kilifi, Kwale and Taita Taveta). These counties were selected because they have high concentrations of KP, high HIV prevalence (and incidence) rates among the general population, and are the focus of existing HIV prevention, care, and treatment efforts.

### *Jilinde’s* conceptual framework

**Figure 2.**
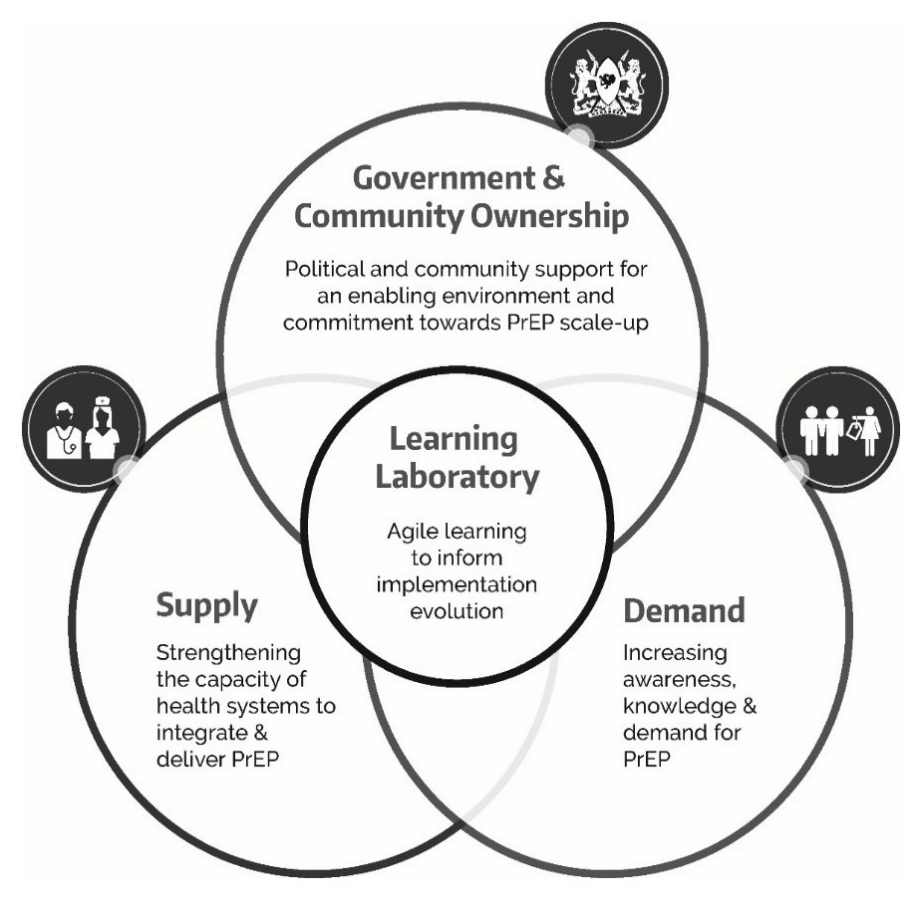
*Jilinde’s* framework for implementation of PrEP at scale in Kenya.

The overlapping domains of supply, demand, and government and community ownership all contribute to a learning laboratory approach to improving implementation of PrEP. Figure 2 outlines this framework showing the interrelatedness of the domains. 

**Figure 3.**
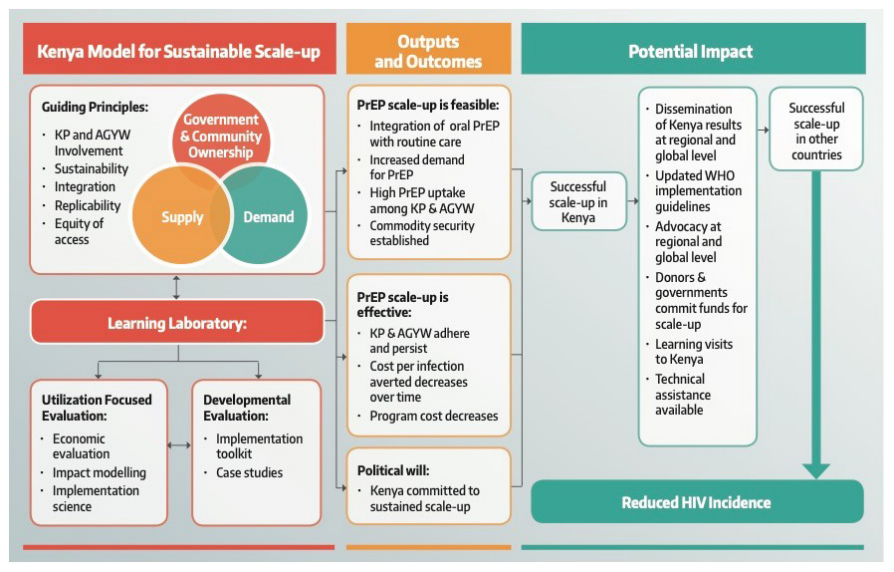
*Jilinde* project theory of change. (KP, key populations; AGYW, adolescent girls and young women; PrEP, pre-exposure prophylaxis).

*Jilinde*’s theory of change is premised on this framework as a model for sustainable PrEP scale-up in Kenya. Implementation of this model should lead to increased political will and demonstrate the feasibility and effectiveness of PrEP scale-up. If successful, this should contribute to successful PrEP scale-up in Kenya. Dissemination of lessons from Kenya should contribute to successful PrEP scale-up in other countries and an overall reduction in HIV incidence in the long-term. The anticipated outcomes and potential impact from this model are summarized in Figure 3.

### Supply of PrEP services

Service delivery is the cornerstone of the *Jilinde* project, which leverages existing health infrastructure, staff, and outreach of existing clinical sites to scale up routine delivery of oral PrEP. The project strengthens clinical sites that were already serving KP and AGYW clients, integrating PrEP into existing combination HIV prevention activities. These clinical sites include: drop-in-centers (DICEs), which are stand-alone clinics that mainly serve KP; public health facilities; and private health facilities. To address access challenges, these sites also provide PrEP services through community delivery models that include safe spaces for AGYW and hotspot outreaches for KP. Service delivery points are being strengthened to provide oral PrEP by ensuring availability of commodities, building health service provider PrEP competency, enhancing referral pathways, and offering outreach services in times and places convenient for KP and AGYW.

Systematic delivery of PrEP clinical services was preceded by engagement meetings with the county health management teams to build a consensus on the implementation agenda. Once county scale-up roadmaps were developed, health facilities were selected by the project team and the county health management team to take part, by way of a facility assessment to establish capacity gaps in providing HIV services and status of infrastructure for delivering PrEP. *Jilinde* supported NASCOP to train a pool of PrEP national master trainers drawn from all 47 counties, who have since cascaded the PrEP training to health service providers within their respective counties, using the nationally approved training curriculum. Additional training focuses on sensitivity in working with KP and AGYW. The trainings are building service providers’ knowledge and skills to initiate and follow up with PrEP clients and increase health service providers’ sensitivity and willingness to work respectfully with KP and AGYW.

The project collaborated with NASCOP to establish a centralized national supply chain for PrEP commodities that leveraged the existing antiretroviral drugs pipeline via the Kenya Medical Supplies Authority (KEMSA). Initial donations of PrEP medication to jumpstart the scale-up were integrated into KEMSA for national-level distribution. To ensure seamless ordering, consumption, and reporting on PrEP commodities, existing logistics management information system tools were revised by NASCOP to include PrEP and then printed and distributed. Health service providers were trained on recording of site-level consumption data and ordering of commodities. DICEs were linked to government-owned health facilities as satellite sites in order to receive PrEP commodities through the national pipeline. An initial “push” order of commodities was initiated by NASCOP to select sites to jumpstart the national scale-up. Subsequently, health facilities shifted to a “pull” system through routine reporting of commodity consumption.

**Figure 4.**
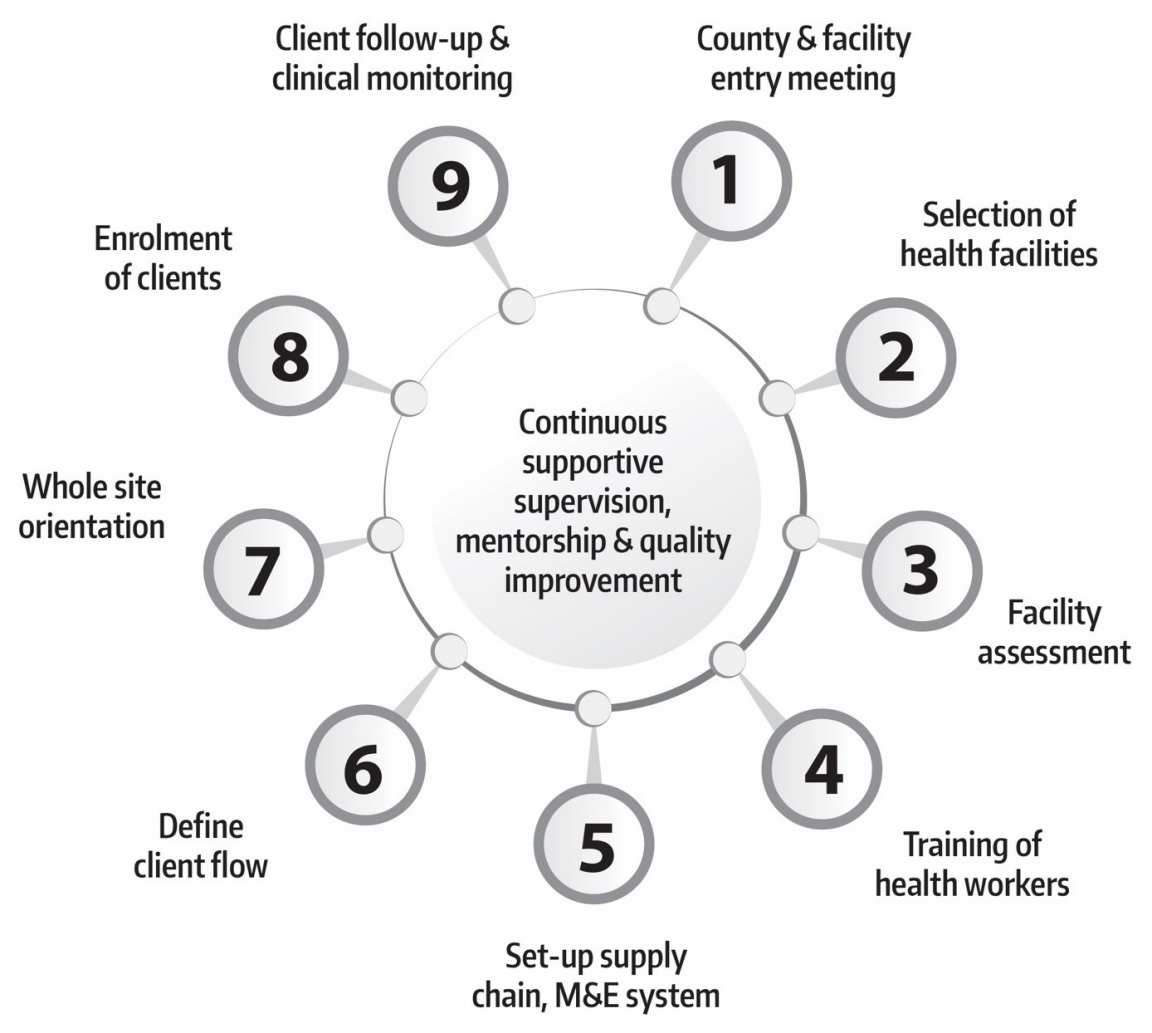
Systematic process of setting up PrEP service delivery sites. (M&E, monitoring and evaluation).

Establishing client flow, especially for the high-volume facilities, was critical, since PrEP clients often needed to visit multiple service points within a facility, including the HIV testing room, clinical diagnosis room, and pharmacy. Whole-site orientations with all staff in the facilities were conducted to build support for the intervention across all cadres within the sites. Health facilities began client enrollment once all these critical steps were implemented. Subsequently, sites also established measures for client follow-up to enhance adherence and continuation. In addition, service providers are offered continuous support and mentorship for quality improvement. Figure 4 summarizes the stepwise process of setting up PrEP services in the clinical sites.

The project is also strengthening the capacity of the county health management teams to provide ongoing supportive supervision and mentoring of trained providers and counselors. This is intended to ensure the retention of knowledge and skills, as well as the maintenance of high-quality services. Periodic supportive supervision visits are enabling sharing of real-life implementation challenges that inform scale-up and case studies that drive changes in approach if needed.

### Demand for PrEP

*Jilinde*’s approach to demand generation is two-fold: use of mass media for raising awareness of PrEP in the general population and targeted communication to the priority populations to generate demand for PrEP, primarily through interpersonal communication. The project has invested in awareness creation for the general population through mass media platforms including television, national and local radio advertising, and talk shows. Furthermore, social media platforms (mainly Facebook, Twitter, and WhatsApp) have been used to facilitate discourse on PrEP. In addition, promotional events and community engagement fora have given visibility to and ignited community conversations on PrEP. This has proven to be important as it provides an environment that enables clients to start and continue using PrEP.

To determine targeted demand creation components, the project is employing innovative methodologies including behavioral economics research, consumer market research, and human-centered design processes to ensure resonance and relevance of messaging. The project is ascertaining end-user perceptions through insights derived from market research and behavioral economics to adequately segment the target audience for demand creation purposes. Through this audience segmentation, the demand creation strategies are adopting a multipronged and multifaceted approach that takes into account the diversity of KPs and AGYW. This has enabled the project to adopt a targeted approach as opposed to a mass “one size fits all” approach. Using human-centered design methodologies, the project developed messages and materials that address the key barriers identified by users themselves. The project used a rapid, iterative process that included pre-testing, piloting, and scaling up of proven interventions. The choice of messengers is also fundamental, and the project is leveraging the robust peer outreach system currently employed as part of the national KP service standards. *Jilinde* is engaging gatekeepers, KP community members, and civil society organizations with strong roots and experience in KP programming to roll out the demand creation activities identified as most promising.

***Adherence support*.** Evidence from clinical trials shows that PrEP effectiveness is dependent on medication adherence; studies reporting high adherence reported high efficacy, while those that reported low adherence reported little or no efficacy ^29– 31^. Given that *Jilinde* is implementing PrEP in routine service delivery, adherence support has been integrated into existing activities through the following approaches:

- **Community-based approaches**: Growing evidence suggests that health outcomes are improved when clients participate in care as part of a supportive cohort and different types of peer support are a mainstay of KP programs ^32, 33^. *Jilinde* is tapping into existing KP and AGYW networks to support both PrEP uptake and adherence, which are inseparable. KP and AGYW networks, support groups, and peer educators who mobilize clients for services play a dual role in ensuring uptake as well as continuity through adherence support and physical tracing of clients lost to follow-up. PrEP clubs, peer support groups, and networks meet periodically in community venues to address adherence challenges and provide peer support. Clients using PrEP are also encouraged to identify support networks of peers or a “buddy” who provide ongoing support to promote adherence through in-person follow-up. The project is also tapping into satisfied PrEP users as PrEP champions, who destigmatize the use of PrEP and establish PrEP use as a social norm among these populations. Given that satisfied clients can be influential proponents for uptake and adherence, some of the early adopters of oral PrEP, who have accepted, enrolled, and continued to take PrEP for a significant period, share their experiences during demand creation activities where they clarify myths and build confidence on PrEP.
- **Health facility-based approaches** *:* PrEP delivery sites have been implementing several client-centered interventions to improve continuation of care. Clients who are enrolled on PrEP and provided consent, receive short message service (SMS) reminders to take pills and upcoming appointments. The popularity of social media platforms, such as Facebook and WhatsApp, with the priority populations has also provided a platform for virtual support groups especially since the start of the COVID-19 pandemic. Active tracing of clients who miss their clinic appointments is done through phone calls by health service providers and in some cases through peer educators. Finally, the project is providing refills in the community as well as empathetic adherence counseling where counselors help clients develop adherence strategies that support them, rather than judging them for failing to adhere or for missing an appointment.

### Community and government ownership

For effective and sustainable oral PrEP scale-up, ownership of the intervention by the community and the government at all levels is critical – institutionalization will help ensure the sustainability of both demand and supply. *Jilinde* is working with the national government through NASCOP’s leadership to formulate and update appropriate policies and guidelines, coordinate national PrEP rollout through strengthened technical working groups, enhance integration and quality of PrEP service delivery, and develop and roll out a national combination prevention communication strategy. Through participation in the quantification and forecasting of PrEP commodity requirements, the project is contributing to the formulation of a long-term national commodity security strategy. At the county level, *Jilinde* is working closely with devolved government stakeholders to prioritize PrEP in work plans, establish coordination structures, effectively integrate PrEP in routine service delivery, and support county leadership in the scale-up efforts.

Successful implementation of the project is dependent on a supportive and enabling policy environment. Keeping these insights in mind, *Jilinde* is collaborating closely with critical stakeholders, influencers, decision-makers, and target beneficiaries such as AGYW and KP networks in the design, implementation, and monitoring of PrEP rollout, informed by a stakeholder engagement plan developed through stakeholder mapping and analysis. Through this plan, key stakeholders – including bar and brothel owners, parents of AGYW, religious leaders, and general community members – are engaged routinely by the project to create an enabling environment for the delivery of PrEP services, thereby creating safe spaces for health promotion events and outreaches for PrEP provision and protecting KP and AGYW on PrEP from harassment. *Jilinde* is using the evidence generated by the project to make decisions on adaptations needed to galvanize support and ownership for sustainability.

### *Jilinde* as a learning laboratory

*Jilinde* is using two evaluation approaches: developmental evaluation (DE) and prospective utilization-focused evaluation (UFE). Participatory DE is being used to guide continuous, iterative program learning through implementation experiences and documentation of learning toward the goal of evolving the implementation approach. The critical feature of DE is the right people reflecting on the right data at the right time and then using those reflections to inform changes in the intervention ^34^. Use of DE is enabling *Jilinde* implementation to be agile, making iterative adjustments to minimize program risks and maximize learning. *Jilinde* is also collecting formative, process, and summative data to answer a set of essential learning questions in a structured and rigorous way. In addition, *Jilinde* is using UFE to help answer specific national oral PrEP research questions outlined in the national framework for the implementation of PrEP in Kenya ^35^. The UFE is focused on understanding client and user experiences, health managers and service provider experiences, community perceptions, user feedback on demand generation, and client acceptability and adherence. *Jilinde* is using this evidence to ensure real-time feedback on the effectiveness of interventions, rapidly identify the need for course corrections or changes in strategy, and enable the sharing of learning with different groups. This learning is essential to inform improvements in routine service delivery and specific recommendations for scale-up of PrEP in Kenya and other countries.

### Costing and modeling

As part of the learning laboratory, *Jilinde* is also assessing the cost of implementing and delivering PrEP from a service delivery perspective and the client perspective. By understanding both the full cost and unit cost of a PrEP program, it will be possible to estimate and project future resource needs associated with scaling up PrEP nationally. Contingent valuation analysis will provide information about the factors that influence the valuation of services by PrEP clients by assessing their maximum willingness to pay for PrEP services. In addition to assessing the costs of oral PrEP to service providers and clients, *Jilinde* is also conducting modeling to estimate the impact and cost-effectiveness of PrEP for different populations in Kenya using the Goals Model ^36^. Modeling of the impact of PrEP services’ use on HIV incidence and costs (e.g., cost of PrEP vs. cost savings due to HIV infections averted) will provide evidence in the Kenyan situation for scale-up, provide specific recommendations on where and how to rapidly take oral PrEP to scale, and what programmatic outcomes to improve to increase epidemic impact and cost-effectiveness.

## Conclusion

The *Jilinde* project is serving as a “learning laboratory,” which informs as well as evaluates programmatic decisions, and is facilitating iterative explorations of different models of PrEP implementation at scale to fit a real-market, low-resource context. Through addressing the critical domains of demand, supply, and government and community ownership of oral PrEP, *Jilinde* is generating critical lessons on the feasibility and effectiveness of scaling up oral PrEP in low- and middle-income countries. By demonstrating significant saturation of the two groups – AGYW and KP – that are widely recognized and targeted in an effort to reduce HIV incidence in most countries, the findings of this model will be broadly applicable to other countries and, ultimately, other target groups within Kenya in a full national scale-up. Subsequently, these lessons may also provide critical learnings that will be applicable to the introduction of similar HIV prevention products and technologies in the future.

## Data availability

No data are associated with this article.
