# Supplementary material for: An implementation model for scaling up oral pre-exposure prophylaxis in Kenya: Jilinde project
Source: Gates Open Res. 2021 Jul 27;5:113. [Version 1] doi: 10.12688/gatesopenres.13342.1 (PMC8669463; doi:10.12688/gatesopenres.13342.1)
Supplement: Peer review history

# Peer review: Reviewer response for version 1

**DOI:** 10.21956/gatesopenres.14585.r31464

**Published:** 2021-12-15

**Authors:** Douglas Krakower, Elizabeth Christian

**Affiliations:** 1 Division of Infectious Diseases, Beth Israel Deaconess Medical Center, Harvard Medical School, Boston, MA, USA

This well-written letter describes a model for implementing and scaling up access to oral PrEP in 10 counties in Kenya where incidence of HIV acquisition is highest, with a focus on reaching the populations with the highest rates of new HIV infections, comprised of adolescent girls and young women, female sex workers, men who have sex with men, people who inject drugs and transgender people. The theory-driven model seeks to engender political and community support for wide-spread access to PrEP, as well as increase demand for PrEP through a variety of public outreach campaigns. It also directly addresses anticipated supply chain issues by using already existing infrastructure and systems, to ensure that as demand for PrEP increases, the medication remains widely available in the community. Significant resources were devoted to supporting PrEP adherence and continuation, including integration of peer support interventions, as well as ongoing assessment and support to service providers to ensure quality care. A system of real-time feedback was used to allow for rapid revision of processes in response to issues as they were identified, which is a major methodological strength for this program.

 This is a thorough approach to large-scale PrEP implementation, and the emphasis on identifying and addressing potential systematic barriers, as well as the high value placed on community engagement, is notable. The emphasis on building patient support for PrEP adherence and continuation is particularly impressive and represents a best-practice in terms of patient-centered health care.  

 The discussion of cost is addressed briefly, and focuses mainly on collecting data about the implementation and service delivery cost, as well as potential cost savings from a reduction in new HIV diagnoses and associated costs. Could the authors clarify how the direct cost to the patients of PrEP medications and associated care was being addressed, as lack of insurance coverage or inadequate insurance coverage for PrEP and associated services has been a major barrier to PrEP roll-out in the US?

 Could the authors also describe any plans to protect confidentiality for adolescents who may be accessing PrEP without their parents' knowledge, especially as adolescent girls experience one of the highest rates of new HIV infections in Kenya? The article describes the provision of safe spaces for adolescents to access care, and efforts to engage parents of adolescent girls and young women, which are strengths. However, the cost of PrEP, as well as the outreach to promote adherence, may be a barrier to girls who don’t want their parents to be aware of their sexual activity. Any additional description of ways to address these challenges would strengthen the manuscript. 

 Please consider the following comments and suggestions for the Introduction:

1. “Substantial work remains to lower HIV transmission rates among these groups, who have a higher prevalence and incidence than the general population, as this high HIV prevalence and incidence threatens the sustainability of gains made in the general population.” Consider revising this sentence to reflect that there is inherent value in decreasing HIV incidence among key groups, and not only to sustain gains in the general population?
2. Consider replacing the term “high-risk individuals” with “individuals at risk for HIV”, and “high-risk sexual encounters” with “sexual encounters associated with HIV exposure” (or similar), as these may represent less stigmatizing language?
3. “...many countries are still skeptical…” Could the authors clarify if this represents viewpoints of national leaders and policymakers or general population opinions, or something else? 

Overall, this letter will be of great interest to public health practitioners and policymakers who seek to scale up PrEP in additional nations and regions, as it offers a clear and compelling description of a multilevel program to improve PrEP use on a national level. If the authors could add any data on the program’s successes with PrEP, even if preliminary, that would further strengthen this important letter.

Does the article adequately reference differing views and opinions?

Yes

Are all factual statements correct, and are statements and arguments made adequately supported by citations?

Yes

Is the Open Letter written in accessible language?

Yes

Where applicable, are recommendations and next steps explained clearly for others to follow?

Yes

Is the rationale for the Open Letter provided in sufficient detail?

Yes

Reviewer Expertise:

Infectious diseases; HIV prevention; preexposure prophylaxis

We confirm that we have read this submission and believe that we have an appropriate level of expertise to confirm that it is of an acceptable scientific standard.

# Peer review: Reviewer response for version 1

**DOI:** 10.21956/gatesopenres.14585.r31465

**Published:** 2021-12-13

**Authors:** David A.M.C. van de Vijver

**Affiliations:** 1 Department of Viroscience, Erasmus MC, Rotterdam, The Netherlands

Were and colleagues introduce the Jilinde project which is a “learning laboratory” approach that informs strategic and continuous learning on implementation and use of PrEP in Kenya. This is an important topic as the roll-out of PrEP has been slow in low- and middle-income countries, despite of the high efficacy of PrEP in preventing HIV. Many countries are sceptical about the feasibility of national rollout of oral PrEP due to a gap in evidence on the feasibility and effectiveness of population-level PrEP interventions in low- and middle-income countries.

 The Jilinde project will implement oral PrEP at scale, while simultaneously deriving lessons to understand the barriers and enablers to PrEP scale-up. The project will be implemented in ten counties in Kenya with a medium or high HIV incidence. The counties also have a high concentration of key populations in whom about one third of all HIV infections in Kenya are reported. Jilinde also pays special emphasis to adolescents and young women that in whom also one third of new infections are found. The emphasis on young women is important due to their reported low adherence to PrEP. The Jilinde project will include adherence support to ensure that individuals at high risk of infection will continue the use of PrEP. The project will also ensure community and government ownership and will perform modelling and costing studies.

 I was impressed by the work that is proposed and looking forward to read about the results of the Jilinde project in the future.

Does the article adequately reference differing views and opinions?

Yes

Are all factual statements correct, and are statements and arguments made adequately supported by citations?

Yes

Is the Open Letter written in accessible language?

Yes

Where applicable, are recommendations and next steps explained clearly for others to follow?

Yes

Is the rationale for the Open Letter provided in sufficient detail?

Yes

Reviewer Expertise:

Infectious disease epidemiology, mathematical modelling and cost-effectiveness, HIV drug resistance, virology

I confirm that I have read this submission and believe that I have an appropriate level of expertise to confirm that it is of an acceptable scientific standard.

# Peer review: Reviewer response for version 1

**DOI:** 10.21956/gatesopenres.14585.r30980

**Published:** 2021-09-13

**Authors:** Deborah J. Donnell

**Affiliations:** 1 Department of Global Health, University of Washington, Seattle, WA, USA; 2 Vaccine and Infectious Disease Division, Fred Hutchinson Cancer Research Center, Seattle, WA, USA

This report details a 6 year effort to implement PrEP at scale in 10 countries in Kenya. The project was ambitious, including in its "Learning laboratory" framework of demand creation, supply chain management and local government and community ownership, a scope incorporating key populations, adolescent girls and young women, public health and private clinics.

 The letter usefully lays out the succession of processes and engagement across the spectrum of entities involved in setting up PrEP for success at scale. No data about the success of the project is yet available, but this is a clear description of the framework, approaches taken in each entity, that will be useful for setting out the steps for success, if the project ultimately demonstrates increase in uptake, persistence of PrEP and good adherence for those persons at high risk of HIV infection. 

 The approach is well justified, and the letter is clearly written, and well organized. Details of the processes are high level and it would be useful if the authors could be sure that references to manuals or other detailed materials developed during the project are available for those interested in learning from this project.

 The authors correctly note that rollout of PrEP, in spite of its known effectiveness, has been slow, and the barriers are complex. This description of the Jilinde project's comprehensive approach to implementation, encompassing skepticism, demand creation, training, logistics, and supply chain, if successful, has the potential to provide a proven road map to PrEP implementation.

Does the article adequately reference differing views and opinions?

Yes

Are all factual statements correct, and are statements and arguments made adequately supported by citations?

Yes

Is the Open Letter written in accessible language?

Yes

Where applicable, are recommendations and next steps explained clearly for others to follow?

Yes

Is the rationale for the Open Letter provided in sufficient detail?

Yes

Reviewer Expertise:

Infectious disease epidemiology, implementation, PrEP, statistics.

I confirm that I have read this submission and believe that I have an appropriate level of expertise to confirm that it is of an acceptable scientific standard.
